# Development of Stock Networks Using Part Mutual Information and Australian Stock Market Data

**DOI:** 10.3390/e22070773

**Published:** 2020-07-15

**Authors:** Yan Yan, Boyao Wu, Tianhai Tian, Hu Zhang

**Affiliations:** 1 School of Mathematics and Physics, Wuhan Institute of Technology, Wuhan 430205, China; yanyan@wit.edu.cn; 2 School of Mathematics, Monash University, Melbourne, VIC 3800, Australia; boyao.wu@monash.edu; 3 School of Statistics and Mathematics, Zhongnan University of Economics and Law, Wuhan 430073, China

**Keywords:** part mutual information, stock relation network, path-consistency, correlation coefficient, clique, degree

## Abstract

Complex network is a powerful tool to discover important information from various types of big data. Although substantial studies have been conducted for the development of stock relation networks, correlation coefficient is dominantly used to measure the relationship between stock pairs. Information theory is much less discussed for this important topic, though mutual information is able to measure nonlinear pairwise relationship. In this work we propose to use part mutual information for developing stock networks. The path-consistency algorithm is used to filter out redundant relationships. Using the Australian stock market data, we develop four stock relation networks using different orders of part mutual information. Compared with the widely used planar maximally filtered graph (PMFG), we can generate networks with cliques of large size. In addition, the large cliques show consistency with the structure of industrial sectors. We also analyze the connectivity and degree distributions of the generated networks. Analysis results suggest that the proposed method is an effective approach to develop stock relation networks using information theory.

## 1. Introduction

A complex network is defined as the system with a large number of node or variables within the system and the relationships between these nodes [1,2,3]. Recent advances in science and technology have generated large-scale networks, such as computer networks, internet networks, gene and protein interaction networks, financial networks, and social networks [4,5]. Network science has become an important method to study the underlying relationship and regulation mechanisms inside the complex systems. With the availability of big datasets, it is imperative to design effective approaches to develop network models for a wide range of complex systems [4,5,6,7,8].

Stock relation network is a rapidly developed research area in recent years due to the availability of observation data [9]. Each stock is a node in this type of network, and the research purpose is to find the potential relationship and influence between stocks using the stock market data [10]. There are three major methods for developing stock networks. The minimum spanning tree (MST) uses the minimal number of edges to construct a network but still maintains the hierarchical structure of the network [11]. The asset graph or threshold value method selects the most important relations to construct stock networks, though a key issue of this method is that the generated asset graph may be disconnected sub-graphs when only a certain number of important relations are selected [12]. The planar maximally filtered graph (PMFG) uses the graph theory to maintain more information in the stock networks [13,14]. Empirical studies suggest that PMFG maintains not only the hierarchical structure of MST but also important relations in the asset graph [15,16]. There are some variations of these methods such as the dynamic spanning tree (DST) [17], triangulated maximally filtered graph (TMGF) [18], p-threshold approach [19], and cointegration analysis [20]. These methods have been used to study the topological structure and performance of the stock markets [21,22,23,24].

The correlation coefficient is the most important measure to evaluate the relationship between stocks [25,26]. However, this measure concentrates on the pairwise relationship only but neglects the influence of the other stocks in the system. To address this issue, partial correlation coefficient has been used to investigate the controlling effects of other stocks on the stock relationship. There are two different approaches to study the controlling effects. The first method investigates the controlling effects of only one or two stocks by using the low order partial correlation [27,28]. The second approach considers the influence of all the other stocks in the system on the considered stock pair. This method then leads to the Gaussian graph model under the assumption of multivariate Gaussian random variable distributions [29].

Compared with correlation coefficient for linear relationships, mutual information (MI) from Shannon’s entropy theory is able to evaluate nonlinear relations and has been used recently to study complex systems and stock networks [30,31]. We have used MI to develop the MST network for the Shanghai Stocks Exchange (SSE) market [32]. MI has also been used to develop the stock network by using the high-frequency data from the Indian stock market [33]. In addition, a number of other approaches have been proposed to use information theory to develop stock networks, including partial mutual information [34], mutual information rate [35], and modified mutual information distance [36]. Although partial mutual information, which is also called conditional mutual information (CMI), has been widely used to develop complex networks in biology, engineering, and physical sciences [37,38,39], this information measure is much less used to construct stock networks. Recently, part mutual information (PMI) is proposed to accurately quantify the nonlinearly direct associations between the measured variables [40].

In this work, we propose to use PMI to measure the relationships between stock pairs, and use the path-consistency algorithm [37,41] to select the significant relationships to construct stock relation networks. This algorithm is efficient for constructing large sparse complex networks and shows robust performance for analyzing the severely contaminated data or heavy outliers [42]. The Australian Stock Exchange (ASX) data are used to examine the effectiveness and performance of our proposed method.

## 2. Methods

This section discusses the information theory for calculating MI, CMI, and PMI. Then we introduce the path-consistency algorithm for constructing stock relation networks, and also discuss MST and PMFG for comparison study.

### 2.1. Information Theory

Instead of analyzing stock prices, we use the log-return of the stock price in this study. The log-return data are assumed to be stationary. Let $P_{i,t}$ be the closing price of stock *i* at trading day *t*. Then the log-return $R_{i,t}$ of this stock at trading day *t* is given by

(1) $$R_{i,t}=\ln\frac{P_{i,t}}{P_{i,t-1}}=\ln P_{i,t}-\ln P_{i,t-1},$$

which represents the relative change of stock prices.

It is assumed that the log-return values defined by (1) are samples of a random variable. Let *X* be a random variable with density function p(x). We first introduce the concept of entropy, which in statistical mechanics is the measure of a system’s thermal energy per unit temperature that is unavailable for doing useful work. The entropy H(X) of *X* is defined by

(2) $$\begin{array}{ccc} H(X) & = & -\sum_{i=1}^{N_{x}}p\left(x_{i}\right)\log p\left(x_{i}\right)\\ H(X) & = & -\int_{\Omega_{X}}p\left(x\right)\log p\left(x\right)dx, \end{array}$$

for discrete and continuous random variables, respectively, where $x_{1},\ldots ,x_{N_{x}}$ are samples of random variable *X* in the discrete case [43]. In addition, for two random variables *X* and *Y*, the joint entropy H(X,Y) is defined by

(3) $$\begin{array}{ccc} H(X,Y) & = & -\sum_{i=1}^{N_{x}}\sum_{j=1}^{N_{y}}p\left(x_{i},y_{j}\right)\log p\left(x_{i},y_{j}\right)\\ H(X,Y) & = & -\int \int_{\Omega_{X}\times \Omega_{Y}}p\left(x,y\right)\log p\left(x,y\right)dxdy, \end{array}$$

for discrete and continuous random variables, respectively, where p(x,y) is the joint density function of random variables *X* and *Y*, and $y_{1},\ldots ,y_{N_{y}}$ are samples of random variable *Y*.

Mutual information (MI) measures the nonlinear dependency between two random variables. For discrete variables *X* and *Y*, MI can be calculated from

(4) $$MI\left(X,Y\right)=\sum_{i=1}^{N_{x}}\sum_{j=1}^{N_{y}}p\left(x_{i},y_{j}\right)\log\frac{p(x_{i},y_{j})}{p\left(x_{i}\right)p\left(y_{j}\right)},$$

where p(x) and p(y) are marginal density functions of variables *X* and *Y*, respectively. In addition, MI can be measured in terms of entropies as

(5) MI(X,Y)=H(X)+H(Y)−H(X,Y).

If the MI value is zero, two random variables are independent of each other [37]. However, a larger value of MI generally suggests closer relationship between random variables. For a system with more random variables, the strong dependent relationship of two random variables may be caused by another random variable. To address this issue, CMI measures conditional dependency between two random variables under the condition of the third variable. The CMI between variables *X* and *Y* given *Z* is defined by

(6) CMI(X,Y|Z)=H(X,Z)+H(Y,Z)−H(Z)−H(X,Y,Z),

where H(X,Y,Z) is the joint entropy of these three random variables, defined by

(7) $$\begin{array}{ccc} H(X,Y,Z) & = & -\sum_{i=1}^{N_{x}}\sum_{j=1}^{N_{y}}\sum_{k=1}^{N_{z}}p\left(x_{i},y_{j},z_{k}\right)\log p\left(x_{i},y_{j},z_{k}\right)\\ H(X,Y,Z) & = & -\int \int \int_{\Omega_{X}\times \Omega_{Y}\times \Omega_{Z}}p\left(x,y,z\right)\log p\left(x,y,z\right)dxdydz, \end{array}$$

and p(x,y,z) is the joint density function of random variables *X*, *Y*, and *Z*, and $z_{1},\ldots ,z_{N_{z}}$ are samples of random variable *Z*.

For discrete random variables, the CMI can also be calculated from

(8) $$\mathrm{CMI}\left(X,Y|Z\right)=-\sum_{i=1}^{N_{x}}\sum_{j=1}^{N_{y}}\sum_{k=1}^{N_{z}}p\left(x_{i},y_{j},z_{k}\right)\log\frac{p(x_{i},y_{j}|z_{k})}{p\left(x_{i}|z_{k}\right)p\left(y_{j}|z_{k}\right)},$$

where p(x|z) and p(y|z) are the density functions of random variables *X* and *Y* under the given third random variable *Z*, respectively, and p(x,y|z) is the joint probability of the random variables *X* and *Y* under the condition of given *Z*. If variables *X* and *Y* are independent of each other under the condition of variable Z, then CMI(X,Y|Z)=0.

For a network of *m* stocks, the log-return of stock $X_{i}$ is measured at different time points $\left(R_{i,1},\ldots ,R_{i,n}\right)$. We can calculate the frequency of the log-return and then use the frequency to approximate the MI [32]. The interval $\left[\min_{j}\left(R_{i,j}\right),\max_{j}\left(R_{i,j}\right)\right]$ is first divided into *k* subintervals, and then compute the frequency of the log-return $f_{i,q}$ falling into the subinterval *q*, and then approximate the probability $p_{i,q}$ by using

(9) $$p_{i,q}\approx \frac{f_{i,q}}{n},\;i=1,\ldots \;m,q=1,\ldots ,k.$$

Similar formulas can be derived for calculating the joint probability p(x,y) of two random variables. Then we can use these approximated probabilities to calculate MI.

We can also calculate MI by using the assumed probability density functions. A particular case is the Gaussian kernel probability density function [37]. The kernel density estimation is a non-parametric method to estimate the probability density function of a random variable. It is a fundamental data smoothing problem where inferences about the population are made. The probability density estimator is given by

$$P\left(X_{i}\right)=\frac{1}{N}\sum_{j=1}^{N}\frac{1}{{\left(2\pi \right)}^{m/2}{\left|C\right|}^{m/2}}\exp\left(-\frac{1}{2}{\left(X_{j}-X_{i}\right)}^{T}C^{-1}\left(X_{j}-X_{i}\right)\right),$$

where *C* is the covariance matrix of variable *X*, |C| is the determinant of matrix *C*, *N* is the number of samples, and *m* is the number of variables in *X*.

Then the entropy of variable *X* can be calculated by

(10) $$H\left(X\right)=\frac{1}{2}m\log\left(2\pi e\right)\left|C\right|,$$

and the MI and CMI are given by

(11) $$\begin{array}{ccc} \mathrm{MI}(X,Y) & = & \frac{1}{2}\log\frac{\left|C(X)||C(Y)\right|}{\left|C(X,Y)\right|}, \end{array}$$

(12) $$\begin{array}{ccc} \mathrm{CMI}(X,Y|Z) & = & \frac{1}{2}\log\frac{\left|C(X,Z)||C(Y,Z)\right|}{\left|C(Z)||C(X,Y,Z)\right|}, \end{array}$$

When using MI to measure the correlation between two variables, the correlation between two random variables is often overestimated, resulting in networks with false positive edges. However, when using CMI to measure the correlation between two variables, the correlation between these two variables is often underestimated, resulting in networks with false negative edges. To address these issues, part mutual information (PMI) is proposed to reduce both the false positive rate and false negative rate [40]. The partial independence of the random variables *X* and *Y* under the given variable *Z* is defined by [40]:

(13) $$p^{*}\left(x|z\right)p^{*}\left(y|z\right)=p\left(x,y|z\right),$$

where

$$p^{*}\left(x|z\right)=\sum_{y}p\left(x|z,y\right)p\left(y\right),$$

$$p^{*}\left(y|z\right)=\sum_{y}p\left(y|z,x\right)p\left(x\right).$$

According to the definition of partial independence Formula (13) and the Kullback–Leibler divergence, PMI is defined by

(14) $$\begin{array}{ccc} \mathrm{PMI}(X,Y|Z) & = & D(p\left(x,y,z\right)||p^{*}\left(x|z\right)p^{*}\left(y|z\right)p\left(z\right)),\\ & = & \sum_{x,y,z}p\left(x,y,z\right)\log\frac{p(x,y,z)}{p^{*}\left(x|z\right)p^{*}\left(y|z\right)p\left(z\right)}, \end{array}$$

where p(x,y,z) is the joint probability distribution of random variables *X*, *Y*, and *Z*, and $D(p\left(x,y,z\right)||p^{*}\left(x|z\right)p^{*}\left(y|z\right)p\left(z\right))$ represents the extended KL divergence from p(x,y,z) to $p^{*}\left(x|z\right)p^{*}\left(y|z\right)p\left(z\right)$. In addition, PMI can also be rewritten as:

(15) $$\mathrm{PMI}\left(X,Y|Z\right)=\sum_{x,y,z}p\left(x,y,z\right)\log\frac{p(x,y|z)}{p^{*}\left(x|z\right)p^{*}\left(y|z\right)}.$$

Based on Formulas (12) and (13), PMI can be further decomposed as follows:

(16) $$\mathrm{PMI}\left(X,Y|Z\right)=\mathrm{CMI}\left(X,Y|Z\right)+D(p\left(x|z\right)||p^{*}\left(x|z\right))+D(p\left(y|z\right)||p^{*}\left(y|z\right)).$$

### 2.2. Path-Consistency Algorithm

Now we consider the problem of constructing stock networks with a number of given stocks. Let G=(V,E) be the graph with node (i.e., stock) set *V* of size |V|=m and edge set *E*. Let $R={\left\{R_{t}\right\}}_{t=1}^{n}$ be the set of samples for the log-return of stock prices. The path-consistency algorithm constructs a graphic network by removing edges that have independent correlation from the fully connected network. To construct a network with adequate sparse structure, a threshold value ϵ>0 is selected, and two stocks are considered as independent correlation if the MI value is less than this threshold.

In the first step, for any adjacent stocks *i* and *j*, if the value of MI(i,j) is less than the threshold, we regard these two stocks are independent, and the edge e(i,j) connecting stocks *i* and *j* is removed from the network. After this step we derive a network whose network density is dependent on the threshold value. This network is called the zero-order PMI network, which is the same network obtained by using the asset graph.

In the second step, we use the first-order PMI to further remove edges from the derived zero-order PMI network. For adjacent stocks *i* and *j* in the zero-order PMI network, we first search for stock *k* that is adjacent to both stocks *i* and *j*. If such stock *k* does not exist, there is no first-order PMI for edge e(i,j), and this edge remains in the network. If one or more stocks exist, we need to calculate one or more first-order PMI values. If the maximal value of these first-order PMI values is less than the threshold value, the edge e(i,j) is removed from the network. This process is applied to all edges in the zero-order PMI network. After this step, we obtain a network that is called the first-order PMI network.

The similar procedure can be applied to the high-order PMI. For example, for the second-order PMI, we need to find two stocks (i.e., stocks $k_{1}$ and $k_{2}$) that all are adjacent to the considered stock pair *i* and *j*, then calculate the second-order $\mathrm{PMI}(i,j|k_{1},k_{2})$, and finally determine whether or not to remove edge e(i,j). After this step, the derived network is called the second-order PMI network. Similarly we can develop high-order PMI networks. It is clear this process must stop at an order of PMI since there are not adequate number of adjacent stocks for calculating higher-order PMI values. Algorithm 1 gives the detailed process of the path-consistency algorithm.
**Algorithm 1** Path-consistency algorithm1:**Input:** the log-return data *R*, significance threshold value ϵ, and complete network G connecting all stocks. 2:For L=0. For each edge e(i,j) in the complete network, calculate the zero-order PMI (i.e., MI(i,j)). Remove this edge e(i,j) by setting G(i,j)=0 if MI(i,j)<ϵ. Then obtain the zero-order PMI network. 3:Let L=L+1. If *L* is larger than the given order, stop the program and output the network *G*
4:For each edge e(i,j) in the (L−1)-order PMI network, search for adjacent stocks connected with both stocks *i* and *j*. Compute the number *T* of these adjacent stocks (not including stocks *i* and *j*). 5:If T<L, stop and edge e(i,j) remains in the network. 6:ElseIf T≥L, select *L* stocks from these T stocks and let them as $K=[k_{1},\cdots ,k_{L}]$, where $k_{L}=C_{T}^{L}$. Compute the L-order PMI(i,j|K) for all $k_{L}$ selections. 7:Find the maximal value of these L-order PMI, denoted as ${\mathrm{PMI}}_{max}\left(i.j|K\right)$. If ${\mathrm{PMI}}_{max}\left(i.j|K\right)<\theta$, remove this edge by setting G(i,j)=0. 8:Return to Step 3. If all edges in the (L−1)-order PMI network are examined, return to Step 2.

### 2.3. Algorithms for MST and PMFG

For comparison studies, we briefly describe the algorithms for constructing MST and PMFG. A MST is the spanning graph with the minimal sum of weights. The MST edge number is m−1 for a network with *m* nodes. This is the minimal number to connect all nodes. Two algorithms are widely used to develop MST, namely the Kruskal algorithm and the Prim algorithm. In this work we use the edges with the large values of MI to develop the MST. To satisfy the requirement of MST, we define the weight between two stocks as $w_{ij}={\mathrm{MI}}_{max}-{\mathrm{MI}}_{ij}$, where ${\mathrm{MI}}_{max}$ is the maximal value of all MI values. This weight is always non-negative and a larger MI value leads to a smaller value of weight. In this way, the problem of finding the large values of MI is changed to search for small values of weight.

The Kruskal algorithm ranks the weights of edges in an ascending order and adds the next edge with the smallest weight if this addition does not create a cycle. The complexity of the Kruskal algorithm is O(mln(m)) where *m* is the number of edges, which is smaller than that of the Prim algorithm. In this work, we use the Kruskal algorithm to construct stock networks. Suppose that G(V,E) is a weighted undirected connective graph with *m* nodes. Algorithm 2 gives the Kruskal algorithm for constructing MST, denoted as T(TV,TE).
**Algorithm 2** Kruskal algorithm for MSTThe set of edges TE is empty, and the set of nodes TV is empty. 2:Rank all edges according to the weight $w_{ij}$ in an ascending order as $E_{1},E_{2},\ldots ,E_{K}$, where K=m(m−1)/2. Add $E_{1}$ into TE and the corresponding two stocks into TV. Set index in as 1. 4:while V−TV is not empty (i.e., size(TV) <m)
Let in=in+1.if $TE+E_{in}$ is cyclic, reject this edge, and go to (a).Elseif (i.e., not cyclic), add edge $E_{in}$ into TE and the corresponding two stocks into TV. Go to Step 3.
End while. Output MST network TE.

The purpose of planar maximally filtered graph (PMFG) is to retain more information. A planar graph is a graph that can be embedded in the plane, i.e., it can be drawn in such a way that no edges cross each other. The edge number of PMFG is 3(m−2). Note that this is still a substantial reduction from the edge number of the complete network, which is m(m−1)/2. The algorithm for constructing PMFG is similar to the algorithm for MST. The difference is, for PMFG, we need to check whether the generated network is still planar. Algorithm 3 gives the detailed process of this algorithm.
**Algorithm 3** Planar maximally filtered graph (PMFG)The set of edges PE is empty, and the set of nodes PV is empty. Rank all edges according to the weight $w_{ij}$ in an ascending order as $E_{1},E_{2},\ldots ,E_{K}$, where K=m(m−1)/2. 3:Add $E_{1}$ into PE and the corresponding two stocks into PV. Set index in as 1. do in=2,…,K
if $PE+E_{in}$ is not planar, reject this edge.Elseif (i.e., it is planar), add edge $E_{in}$ into PE and the corresponding two stocks into PV. Go to Step 3.
End while. **Output:** PMFG network PE.

## 3. Results

In this section we first describe the Australian stock market data. Then we use the path-consistency algorithm to develop four PMI networks with different orders. For comparison study, we also develop the MST and PMFG networks. We then analyze the topological properties of these developed networks.

### 3.1. Stock Market Data

We use the Australian ASX200 daily trading data over the time period from 1 July 2016 to 30 June 2017 (253 trading days) to develop the stock networks. The ASX 200 consists of the 200 largest stocks and accounts for about 82% of the Australian share market capitalization. This index is a capitalization weighted and float-adjusted stock market index listed on the Australian Securities Exchange. Data are download from Yahoo Finance (https://au.finance.yahoo.com/). Six stocks (i.e., MTR, BTT, DHG, CLW, VVR, and WFD) are removed from our analysis because of the incomplete information in the considered time period.

We use MATLAB function *jbtest.m* to test whether the observation data of each stock follow a Gaussian distribution. Function *jbtest.m* performs the Jarque–Bera goodness-of-fit test of composite normality, i.e., that the data come from an unspecified normal distribution. For the stock price data and log-return data of the 194 stocks, there are only 36 and 12 stocks, respectively, for whom the null hypothesis (i.e., the data are normally distributed) cannot be rejected at the 5% significance level. These results suggest that the frequency probability (9) would be used to calculate the MI values when using either the stock price data or the log-return data.

### 3.2. Stock Networks

Using the log-return of the stock price data, we first construct stock networks based on different PMI orders. The first question is the influence of PMI order and threshold value on the developed network structure. We first use the path-consistency algorithm to develop stock relation networks based on different PMI orders and different threshold values. Table 1 gives the edge numbers of generated networks. When ϵ=0.02, there is a large number of edges in the zero-order PMI network. Although a large number of edges are removed from the network by applying the high-order PMI, the edge number in the nine-order PMI network is still 940 and the application of ten-order PMI does not remove any edge from the network. This observation suggests that we cannot expect the application of high-order PMI to generate a sparse network since it is difficult to find enough adjacent stocks to form high-order PMI in the framework of the path-consistency algorithm. On the other hand, it is more practical to use a relatively large threshold value to construct a low-order PMI network to achieve a given network density.

Table 1 suggests that more than 80% of the removed edges from the zero-order PMI network are conducted during the application of the one-order PMI and two-order PMI unless the threshold value is quite small (i.e., λ=0.02). In addition, more than 90% of removed edges from the zero-order PMI network are conducted in the application of the first three orders of PMI. Thus, it is suggested to use the two-order PMI or three-order PMI to construct stock relationship networks, which is consistent with the suggestion in [37].

**Figure 1:**
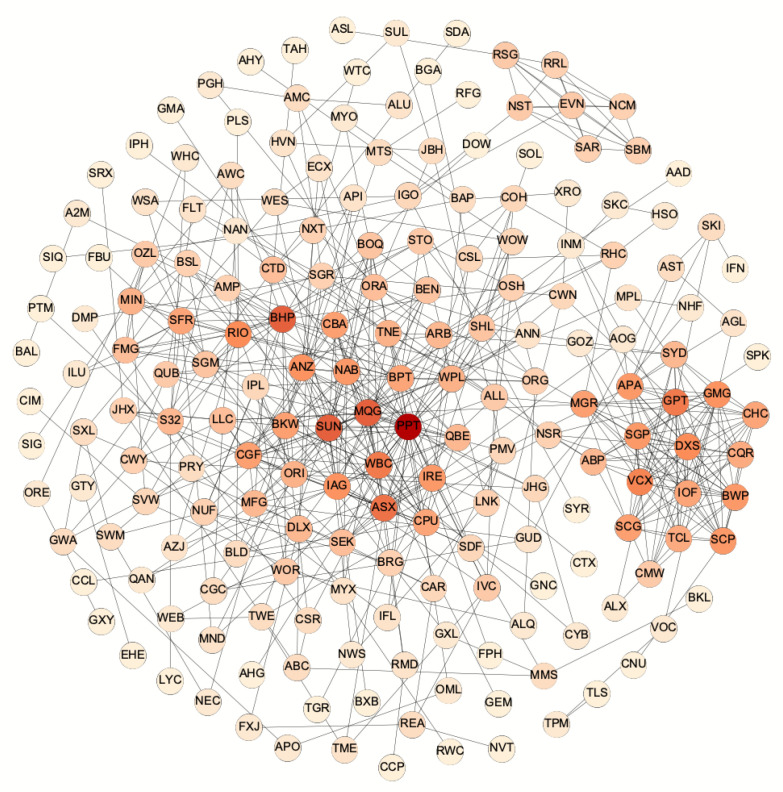
Stock relation Network of the Australian ASX200 by using the second-order PMI. The threshold value is ϵ=0.0477 and edge number is 574. For stocks that have larger numbers of edges (i.e., larger values of degree), the color of the stocks is darker.

**Figure 2:**
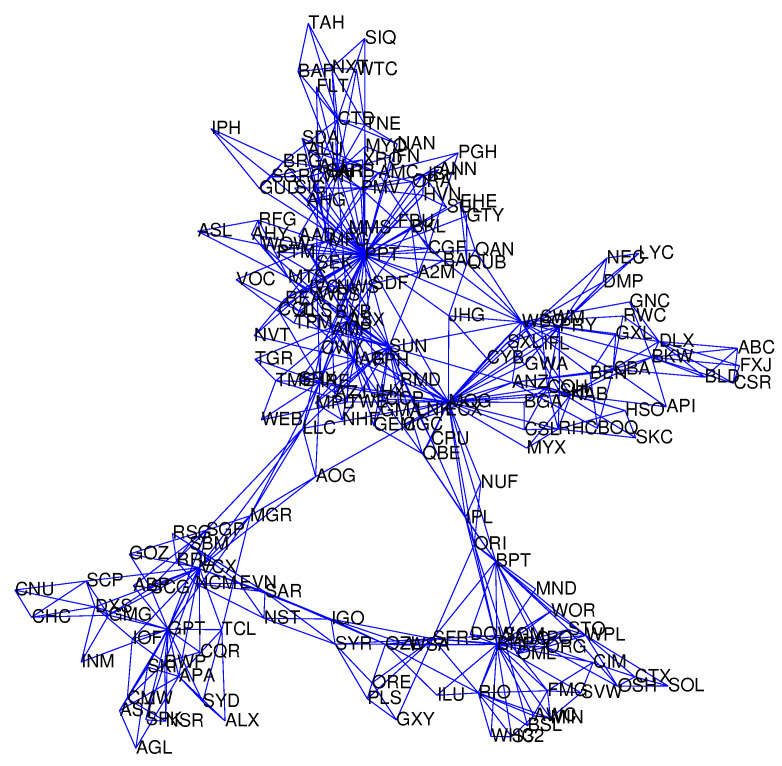
Stock relationship Network of the Australian ASX200 by planar maximally filtered graph (PMFG).

To ensure the developed network is fully connected, we consider networks with an adequate number of edges. Thus, in this work we compare our developed network with the PMFG network whose edge number is 576 (3m−6=3×194−6). The threshold value is selected in order that the edge numbers in the developed networks are around 576. Figure 1 gives the developed two-order PMI stock network using a threshold value λ=0.0477. The edge number of this network is 574. For comparison study, we also develop the PMFG network in Figure 2 by using the developed software in [44]. We also develop the zero-order PMI stock network in Supplementary Figure S1 with 576 edges by using a threshold value λ=0.0832, one-order PMI network in Supplementary Figure S2 with 577 edges by using a threshold value λ=0.062, and three-order PMI network in Supplementary Figure S3 with 576 edges by using a threshold value λ=0.041. In addition, we develop the MST network using the Kruskal algorithm.

For the networks using different orders of PMI, we compare the similarity between these networks. For each pair of networks, we use the network with a smaller number of edges as the standard network. The similarity score is the percentages of the shared edges in these two networks to the edge number of the standard network. Table 2 gives the similarity scores of these four PMI networks, MST and PMFG networks. When the PIM orders of two networks are closer to each other, the value of the similarity score is larger. In addition, the score between the PMI network and MST network is larger if the PMI order is larger. However, the similarity score is only slightly above 50% between the PMFG and any PMI networks, which suggests that there are substantial differences between the PMFG network and the PMI networks.

An interesting question is what edges are removed from the network when a high order PMI is applied to reduce the network density. Table 3 gives the information in the developed PMI networks. Here an edge connecting stocks in the same sector is called the edge of the same sector, while an edge connecting stocks in different sectors is called the edge of different sectors. It shows that, when a higher order of PMI is applied, the proportion of removed edges of different sectors increases. For the detailed numbers for the removed edges in different stages (i.e., PMI order in the same algorithm), the majority of edges of different sectors are removed at the lower orders. When the stage number increases, the proportion of edges of the same sector also increases. For the three-order PMI network, the proportion of edges of the same sector in three-order PMI is even larger than 50%. However, this result is not critical since the number of removed edges in this order is only ∼9% of the total removed edges in three orders together.

### 3.3. Analysis of Cliques

We have developed four networks by using the proposed PMI algorithm with different orders. Next, we analyze the information of cliques in the generated networks. A clique in undirected graphs is a maximal complete subgraph of three or more node. It represents the close relationship between the stocks in a clique. Table 4 gives the number of cliques with different orders in the four generated networks. The clique number in the PMFG network is not presented since it has only 3-clique and 4-clique. Table 4 suggests that it is more possible to generate higher-order cliques in the zero-order PMI network. This result is reasonable since the application of higher-order PMI will remove the redundant edges that are influenced by other stocks. Thus the edges in cliques may be removed when applying the higher-order PMI. However, there are still cliques of orders 12 and 10 in the one-order PMI networks and two-order PMI network, respectively.

We next examine the similarities of the stocks in each clique, namely whether the stocks in a clique are in the same industrial sector. Supplementary Figure S1 suggests that stocks in high-order cliques (order ≥5) show strong similarities in sectors and form three communities in the zero-order PMI network, namely the energy and materials (RM) sector, the financials (FIN) sector, and the real estate (RE) sector. Similar observations can also be found in the other PMI networks. Table 5 gives one clique with the highest order in each sector for each PMI network. For each network, the real estate sector always has cliques with the largest order, and the financial sector follows. These examples show that nearly all the cliques have stocks in the same sector. Certainly, not all cliques in these networks have such high similarity. There are lower order cliques in these networks that have one or two stocks from other sectors.

### 3.4. Network Topology

After studying the similarities between the developed networks, we next examine the topological properties of the developed networks. The first property is the degree distribution. The degree of a node is the number of connections it has to other nodes. The degree distribution is defined as $P\left(k\right)=n_{k}/n$, if there are *n* nodes in a network and $n_{k}$ of them have degree *k*. The degree distribution is very important in studying both real networks and theoretical networks. An important type of networks is the scale-free network whose distribution approximately follows the power low [45]. However, recently studies suggested that most of the observed networks may have fat-tailed degree distribution [46].

**Figure 3:**
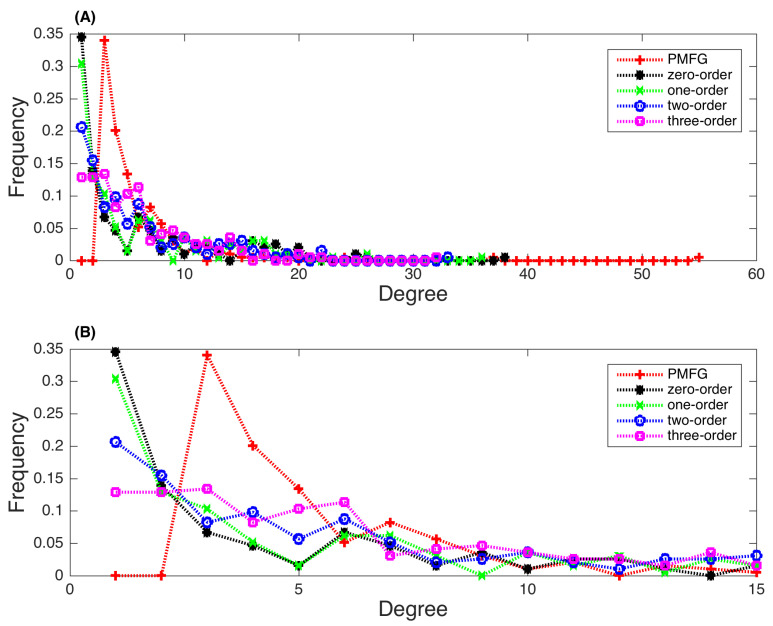
Distributions of degrees in the generated stock networks. (**A**) Distributions for the degrees of all values. (**B**) Detailed distributions for the degrees ranging from 1 to 15. (Red plus: PMFG network; Black star: zero-order PMI network; Green X: one-order PMI network; Blue circle: two-order PMI network; Magenta square: three-order PMI network).

Figure 3A gives the degree distributions of five different networks in full scale, and Figure 3B presents the detailed distributions for the degrees ranging from 1 to 15. The maximal degrees in these five networks are 55, 38, 36, 33, and 32 for the PMFG, zero-order, one-order, two-order, and three-order PMI networks, respectively. The maximal degree is less if a higher-order PMI is applied. The minimal degree in the PMFG network is 3 and then the degree decreases gradually. In the four PMI networks, the degrees follow a power-law distribution. When the PMI order is larger, the network degrees are more relatively evenly distributed. We have calculated the power law for the degree distribution, namely

(17) $$P\left(k\right)=\alpha k^{-\gamma }.$$

Note that for the PMFG network, since the minimal degree is 3, the value of *k* is replaced by k−2. Our results show that the values of γ is 2.075, 1.257, 1.08, 0.82, and 0.71 for the PMFG, zero-order, one-order, two-order, and three-order PMI networks, respectively.

We next study the property of shortest paths in the developed networks. A shortest path, or geodesic path, between two nodes is a path with the minimum number of edges. The length of a geodesic path is called geodesic distance or shortest distance. Geodesic paths are not necessarily unique, but the geodesic distance is well-defined. One of the most discussed network phenomena is the small-world effect, since in most real networks the typical geodesic distance is surprisingly short [47]. The average geodesic distance and the distribution of distances can be used to illustrate the dynamics of a rumor (or a disease) spreading over a social network, effects of collaborations in a research community, and regulatory mechanisms in biological networks [48].

**Figure 4:**
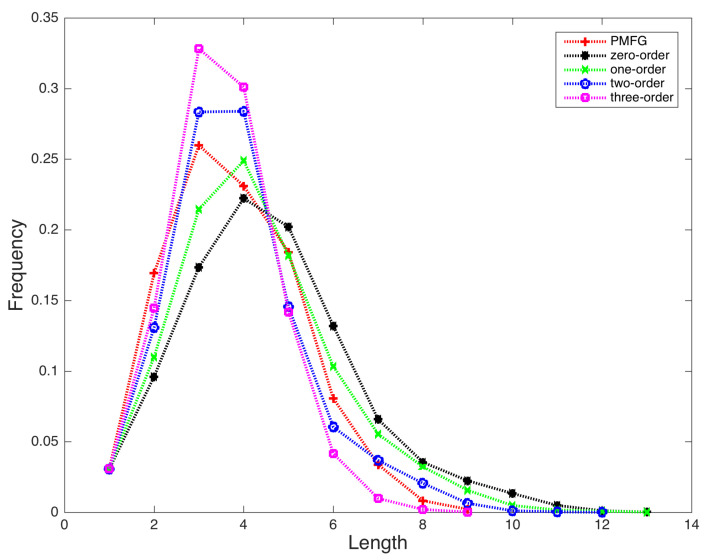
Distributions of lengths in the generated stock relation networks. (Red plus: PMFG network; Black star: zero-order PMI network; Green X: one-order PMI network; Blue circle: two-order PMI network; Magenta square: three-order PMI network).

Figure 4 presents the distributions of lengths for the shortest path connecting the node pairs in the five generated networks. The longest length is 9, 13, 13, 12, and 9 in the PMFG, zero-order, one-order, two-order, and three-order PMI networks, respectively. Basically, the majority of the shortest paths concentrate on paths with 3 or 4 nodes for these five networks. Except for the zero-order PMI network, the other four networks have the largest proportions of the shortest paths with lengths 3 and 4. When a higher order PMI is used, less number of node pairs have long shortest paths. We calculated the mean path length of these networks, defined by

(18) $$\mathrm{ML}=\frac{1}{N(N-1)}\sum_{i\neq j}d_{ij}$$

where $d_{ij}$ is the length of the shortest path between nodes *i* and *j*. The mean path length is 3.80, 4.59, 4.28, 3.86, and 3.55 for the PMFG, zero-order, one-order, two-order, and three-order PMI networks, respectively. These results are consistent with the observations in Figure 4.

## 4. Discussion

This research finds the importantly closed relationships between the three major subsets in the Australian Stock Exchange, namely the real estate subset, financial subset, and energy and materials subset. The real estate subset is denser than the other two subsets because it involves more high-order cliques and the 13-clique and 12-cliques only exist in this subset. This community includes 16 stocks from the real estate sector (i.e., ABP, BWP, CHC, CQR, CMW, DXS, GMG, GPT, GOZ, IOF, MGR, NSR, SCP, SCG, SGP, and VCX), two stocks from the Industrials sector (i.e., SYD and TCL) and two stocks from the Utility sector (i.e., APA and SKI). Stocks DXS, GPT, and VCX have the highest degree at 20. Both DXS and VCX are real estate investment trusts. The DXS directly owns $15.6 billion of office and industrial properties and manages a further $16.2 billion of office, retail, industrial, and healthcare properties for the third party capital partners. The VCX specializes in ownership and management of Australian shopping centers valued approximately at $6.9 billion. The GPT is one of Australia’s largest diversified listed property groups in a portfolio valued at more than $20 billion that includes retail, office and logistics and business park assets. In addition, the SYD owns the Sydney airport, and the TCL is a road operator company that manages and develops urban toll road networks in Australia. Both SYD and TCL are important parts of the Australian transportation system and benefit the development of real estate companies. Moreover, APA is the largest natural gas infrastructure business in Australia, and SKI owns and manages a portfolio of electricity infrastructure assets. Both companies provide essential services for real estate companies.

The financial subset comprises of 21 stocks from six sectors. Among these securities, sixteen of them (i.e., AMP, ANZ, ASX, BOQ, BEN, CGF, CBA, IAG, MQG, MFG, NAB, PPT, QBE, SDF, SUN, and WBC) belong to the Financials sector while the rest stocks come from five different areas (i.e., SHL in the health care sector, QUB in the industrials sector, CPU in the information technology sector, BKM in the materials sector, and LLC in the real estate sector). Among them, stocks PPT, MQG, and SUN are three stocks with the highest degrees in this community (i.e., 38, 26, and 25, respectively), followed by the “big four banks” in Australia (namely NAB, CBA, ANZ, and WBC) and Australia Securities Exchange (ASX). The PPT provides all types of financial advise for a broad range of institutions. The MQG is the world’s largest infrastructure asset manager and the top mergers and acquisitions adviser in Australia. The SUN is a mid-size bank and the largest general insurance group. As one of the largest medical companies listed on ASX 50, the SHL mainly uses the acquisition strategy to expand its business, forming a 6-clique with MQG, ASX, SUN, NAB, and WBC. The CPU is an Australian stock transfer company, offering a series of solutions to financial institutions. The BKW produces a variety of building materials, and the LLC is a multinational construction, property and infrastructure company. Since the big four banks hold about 80% of the home loan market in Australia, it is very likely to find indirect interactions among these two stocks and four big banks.

The energy and materials subset is the largest community in the network, including 28 stocks. There are six stocks in the Energy sector (i.e., BPT, OSH, ORG, STO, WPL, and WOR), one in the Industrials sector (namely MND), and 21 in the materials sector (i.e., AWC, BHP, BSL, EVN, FMG, ILU, IGO, MIN, NCM, NST, ORI, OZL, RRL, RSG, RIO, SFR, SAR, SGM, S32, SBM, and WSA). In this community, stocks BHP, RIO, and BPT have the highest degrees at 25, 19, and 18, respectively. The BHP and RIO are the first- and second-largest metals and mining corporations in the world, and the BPT is the largest onshore oil producer in Australia. The closed connections between material stocks derive from the dominant role played by the material in Australia revenue. Abundant mineral resources in this country stimulate the rapid progress in related companies and lead to similar behaviors in the stock market. There are also deep interactions between material and energy stocks in the energy and materials subset because their corresponding companies are in the same production chain. For example, petroleum is the key business in the BHP, and it forms a 6-clique with five energy companies (i.e., OSH, ORG, STO, WPL, and WOR). As an Australian guide company engaged in oil and gas exploration and production, the BPT, together with some material stocks, consists of several high-order cliques in the PMI network. In addition, the MND from the industrial sector acts as a unique role in this community since it is a leading engineering group providing construction and maintenance services to the resources and energy sectors.

## 5. Conclusions

In this work, we propose to use the information theory to develop stock relation networks. In addition to mutual information that is able to measure nonlinear relationships between random variables, we further propose to use the part mutual information to filter out redundant links between stock pairs. The AXS200 stock market dataset is used to examine the effectiveness of our proposed method. Using the network size of the PMGF network as the standard, we develop four networks using the 0∼3 orders of PMI measures. We compare the similarity and difference between these four PMI networks, MST and PMFG networks, and also compare the topological properties of these four networks with the PMFG network. The degree distributions of the four PMI networks follow the power law, though the value of the power is not large. In addition, if the PMI order is larger, the average length of the shortest paths in the PMI network is shorter.

Compared with the MST and PMFG networks, the threshold method includes edges with the higher correlation relationship based on the same number of edges in the network. The disadvantage of this method is that it may generate separated subgraphs. If we connect all nodes in the network, a smaller threshold value may be needed and the edge number in the network may be quite large. The application of PMI may reduce the network density by removing the redundant edges. However, the disconnection issue in this type of algorithm still remains. It is interesting to design a modified method that is able to include edges with high correlation relationships but also maintain the connection of all nodes. This will be an interesting topic for potential future studies.

## Figures and Tables

**Table 1:** Edge numbers of part mutual information (PMI) networks by using different threshold values λ and different PMI orders.

λ	0-Order	1-Order	2-Order	3-Order	4-Order	5-Order	6-Order	7-Order	8/9-Order
0.02	6227	4399	3012	2004	1422	1140	10214	973	940
0.03	3818	2300	1314	877	692	687	685	682	
0.04	2386	1269	772	599	544	539	538		
0.05	1497	807	535	445	422	418			
0.06	1050	600	440	380	364				
0.07	775	473	365	321	313				
0.08	616	385	301	275	272				

**Table 2:** Similarity scores of the generated networks by using different methods.

Methods	0-Order PMI	1-Order PMI	2-Order PMI	3-Order PMI	MST	PMFG
Zero-order PMI	1	0.8976	0.7491	0.6615	0.8290	0.5417
One-order PMI		1	0.7840	0.6997	0.8549	0.5347
Two-order PMI			1	0.6864	0.8497	0.5436
Three-order PMI				1	0.8860	0.5412

**Table 3:** Information of removed edges in the high order PMI networks. Each set of data includes (number of edges of same sector, number of edges of different sectors, proportion of removed edges of different sectors).

Network\PMI Order	PMI-1	PMI-2	PMI-3	Total
One-order PMI	(126, 284, 0.69)			(126, 284, 0.69)
Two-order PMI	(180, 624, 0.78)	(112, 197, 0.64)		(292, 821, 0.74)
Three-order PMI	(194, 895, 0.82)	(104, 365, 0.77)	(91, 68, 0.42)	(389, 1328, 0.77)

**Table 4:** Number of cliques of different orders.

Clique Order	3	4	5	6	7	8	9	10	11	12	13
Zero-oder PMI	26	14	7	9	10	8	4	3	3	3	1
One-order PMI	19	10	8	9	11	6	2	4	3	2	0
Two-order PMI	34	12	13	9	9	6	6	2	0	0	0
Three-order PMI	50	17	7	6	8	3	0	0	0	0	0

**Table 5:** Information of high order cliques in the generated networks. RE: real estate sector. FIN: financial sector. EM: energy and materials sector. IND: industrial sector.

Size	Network	Stock Names	Sectors
13	0-order PMI	TCL BWP CHC CQR DXS GMG GPT IOF MGR SCP SCG SGP VCX	RE (12), IND (1)
11	0-order PMI	ANZ ASX CGF CBA IAG MQG NAB PPT QBE SUN WBC	FIN (11)
8	0-order PMI	AWC BHP FMG MIN OZL RIO SFR SGM	EM (8)
12	1-order PMI	BWP CHC CQR DXS GMG GPT IOF MGR SCP SCG SGP VCX	RE (12)
11	1-order PMI	ANZ ASX BEN CBA IAG MQG NAB PPT QBE SUN WBC	FIN (11)
8	1-order PMI	AWC BHP FMG MIN OZL RIO SFR SGM	EM (8)
10	2-order PMI	CHC DXS GMG GPT IOF MGR SCP SCG SGP VCX	RE (10)
9	2-order PMI	ANZ CGF CBA IAG MQG NAB PPT SUN WBC	FIN (9)
6	2-order PMI	BHP FMG MIN RIO SFR SGM	MA (6)
8	3-order PMI	CHC DXS GMG GPT IOF SCP SGP VCX	RE (8)
8	3-order PMI	ANZ CGF CBA IAG MQG PPT SUN WBC	FIN (8)
6	3-order PMI	EVN NCM NST RRL SAR SBM	EM (6)

## Supplementary Materials

The following are available online at https://www.mdpi.com/1099-4300/22/7/773/s1, Figure S1: Stock relationship Network of the Australian ASX200 by using the zero-order PMI path-consistency algorithm. The threshold value is λ=0.0832. The edge number is 576. For stocks that have larger numbers of edges (i.e., larger values of degree), the color of the stocks is darker, Figure S2: Stock relationship Network of the Australian ASX200 by using the first-order part mutual information algorithm. The threshold value is λ=0.062. The edge number is 577. For stocks that have larger numbers of edges (i.e., larger values of degree), the color of the stocks is darker, Figure S3: Stock relationship Network of the Australian ASX200 by using the third-order part mutual information algorithm. The threshold value is λ=0.041. The edge number is 576. For stocks that have larger numbers of edges (i.e., larger values of degree), the color of the stocks is darker.

## Abbreviations

The following abbreviations are used in this manuscript:

MST	Minimum spanning tree
AG	Asset Graph
PMFG	Planar maximally filtered graph
MI	Mutual Information
CMI	Conditional mutual information
PMI	Part mutual information
