# Supplementary material for: Development of Stock Networks Using Part Mutual Information and Australian Stock Market Data
Source: Entropy (Basel). 2020 Jul 15;22(7):773. doi: 10.3390/e22070773 (PMC7517323; doi:10.3390/e22070773)
Supplement: Supplementary file 1 [file entropy-22-00773-s001.pdf]

entropy

MDPI

Article

Development of Stock Networks Using Part Mutual Information and Australian Stock Market Data

Yan Yan [1], Boyao Wu [2], Tianhai Tian [2,*], and Hu Zhang [3,*]

1 School of Mathematics and Physics, Wuhan Institute of Technology, Wuhan, China; yanyan@wit.edu.cn
2 School of Mathematics, Monash University, Melbourne, Australia; boyao.wu@monash.edu
3 School of Statistics and Mathematics, Zhongnan University of Economics and Law, Wuhan, China
* Correspondence: zhh11497@sina.com, +86-27-88385285 (H.Z.); tianhai.tian@monash.edu; Tel.: +61-3-99054474 (T.T.)

Supplementary Information

Development of Stock Relationship Networks using Part Mutual Information

S1. Developed networks

This Supplementary Information gives three generated stock networks, namely zero-order PMI network, one-order PMI network, and three-order PMI network.

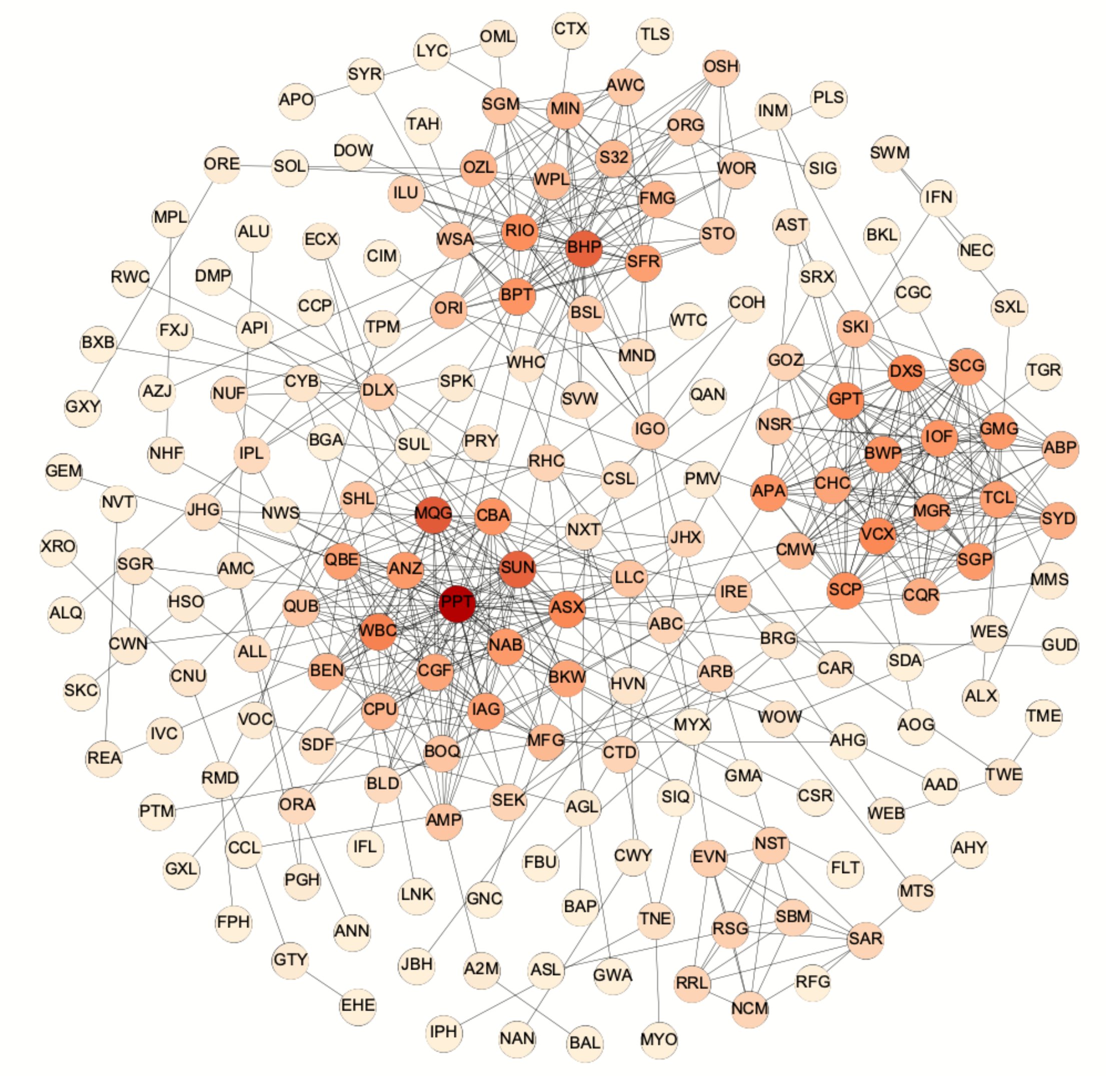

Figure S1. Stock relationship Network of the Australian ASX200 by using the zero-order PMI path-consistency algorithm. The threshold value is $\lambda = 0.0832$. The edge number is 576. For stocks that have larger numbers of edges (i.e. larger values of degree), the color of the stocks is darker.

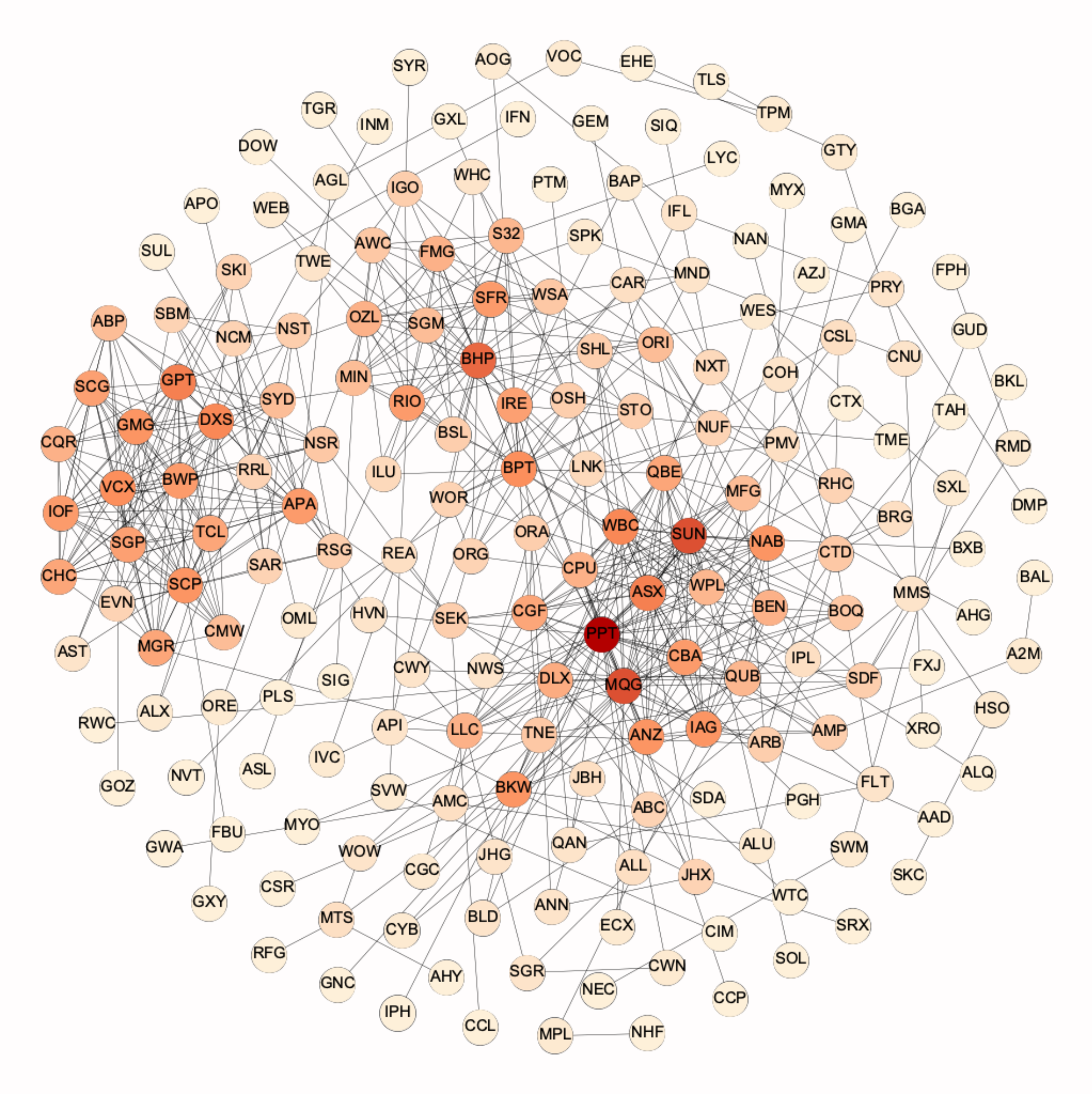

Figure S2. Stock relationship Network of the Australian ASX200 by using the first-order part mutual information algorithm. The threshold value is $\lambda = 0.062$. The edge number is 577. For stocks that have larger numbers of edges (i.e. larger values of degree), the color of the stocks is darker.

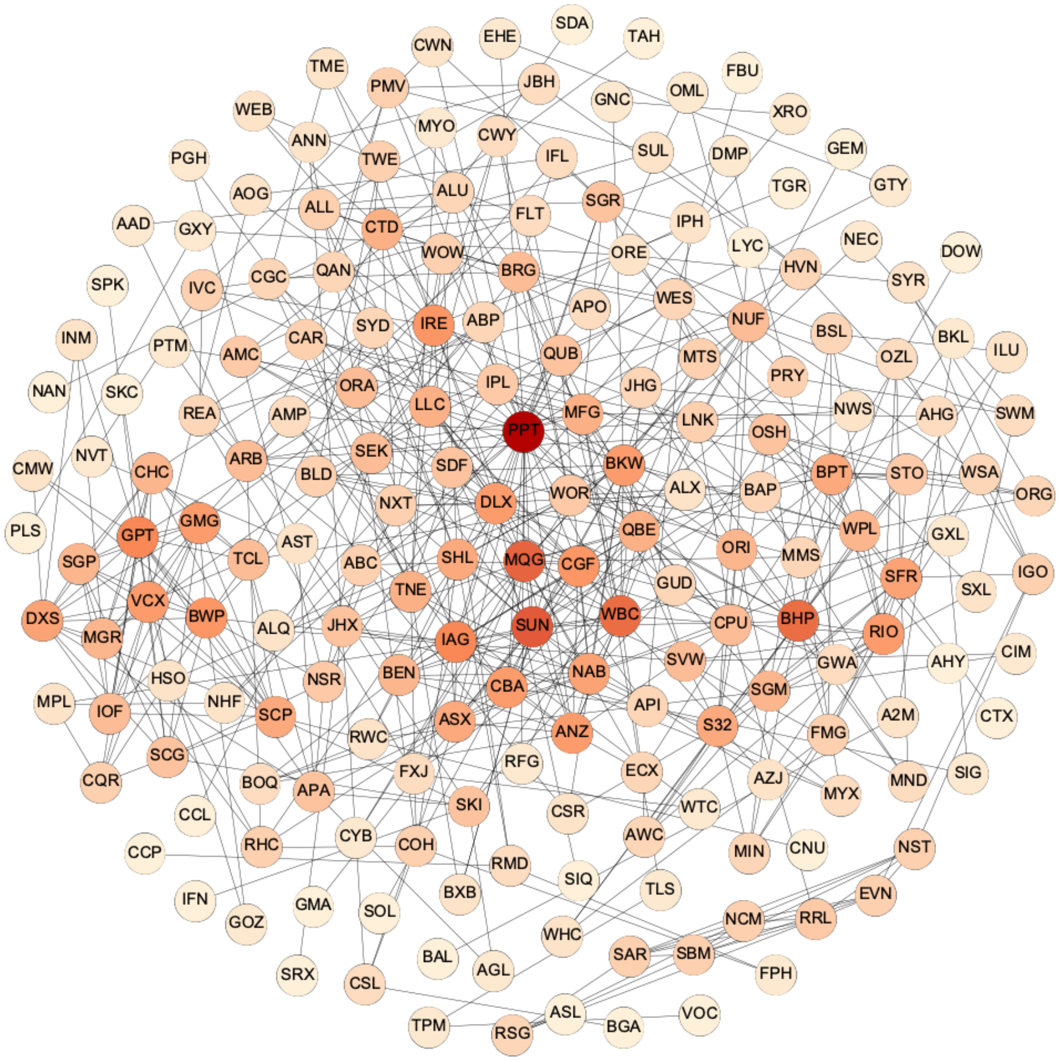

Figure S3. Stock relationship Network of the Australian ASX200 by using the third-order part mutual information algorithm. The threshold value is $\lambda = 0.041$. The edge number is 576. For stocks that have larger numbers of edges (i.e. larger values of degree), the color of the stocks is darker.

© 2020 by the authors. Licensee MDPI, Basel, Switzerland. This article is an open access article distributed under the terms and conditions of the Creative Commons Attribution (CC BY) license (http://creativecommons.org/licenses/by/4.0/).
